# In Vitro Antioxidant and Antiaging Activities of Collagen and Its Hydrolysate from Mackerel Scad Skin (*Decapterus macarellus*)

**DOI:** 10.3390/md20080516

**Published:** 2022-08-13

**Authors:** Elisa Herawati, Yochidamai Akhsanitaqwim, Pipin Agnesia, Shanti Listyawati, Artini Pangastuti, Adi Ratriyanto

**Affiliations:** 1 Department of Biology, Faculty of Mathematics and Natural Sciences, Universitas Sebelas Maret, Surakarta 57126, Indonesia; 2 Department of Animal Science, Faculty of Agriculture, Universitas Sebelas Maret, Surakarta 57126, Indonesia

**Keywords:** antiaging, antioxidant, collagen, hydrolyzed collagen, mackerel scad skin

## Abstract

The skin of mackerel scad fish (*Decapterus macarellus*) is a new source for pepsin-soluble collagen and its hydrolysate, both of which have never been explored. This study aims to characterize and determine the in vitro antioxidant, antiglycation, and antityrosinase activity of pepsin-soluble collagen (PSC) and hydrolyzed collagen (HC) from mackerel scad skin. PSC was extracted using 0.5 M acetic acid containing 0.1% pepsin for 48 h at 4 °C. The obtained PSC was then hydrolyzed with collagenase type II (6250 U/g) to produce HC. The PSC yield obtained was 6.39 ± 0.97%, with a pH of 6.76 ± 0.18, while the HC yield was 96% from PSC. SDS-PAGE and Fourier Transform Infrared (FTIR) analysis showed the typical features of type I collagen. HC demonstrated high solubility (66.75–100%) throughout the entire pH range (1–10). The PSC and HC from mackerel scad skin showed antioxidant activity against 2,2-diphenyl-1-picrylhydrazyl (DPPH), with IC50 values of 148.55 ± 3.14 ppm and 34.966 ± 0.518 ppm, respectively. In the antiglycation test, PSC had an IC50 value of 239.29 ± 15.67 ppm, while HC had an IC50 of 68.43 ± 0.44 ppm. PSC also exhibited antityrosinase activity, with IC50 values of 234.66 ± 0.185 ppm (on the L-DOPA substrate), while HC had an IC50 value of 79.35 ± 0.5 ppm. Taken together, these results suggest that the skin of mackerel scad fish has potential antiaging properties and can be further developed for pharmaceutical and cosmetic purposes.

## 1. Introduction

Every country is experiencing population growth and an increase in the number of elderly individuals. One out of every six people on earth will be 60 or older by 2030. Although the trend of population aging originated in high-income countries, low- and middle-income countries are currently experiencing the most significant changes. By 2050, two-thirds of the world’s population over 60 will be living in low- and middle-income countries [1]. As a middle-income country, Indonesia has an age structure gradually shifting toward more people in older age groups, as evidenced by a rise in the number of people aged 60 and up, which will rise from 18.1 million in 2010 to an estimated 48.2 million by 2035 [2].

Aging is a multifaceted phenomenon marked by both a gradual decrease in the quality of physiological and biochemical processes and an increased susceptibility to disease. The skin is the body’s most voluminous organ, and exhibits the most visible indications of aging, which become more prominent as a person becomes older. Skin aging, like any other pathologic condition, can have a physical and psychological impact on those affected [3,4]. As the main constituent structure of the skin, collagen type I and III decline in their amount by 1% annually. Collagen degradation is also prominent in inflammatory skin diseases and photoaging. Here, the skin becomes thin, dry, hyper/hypopigmented, with the elasticity decreasing and fine lines/wrinkles starting to appear. Thus, there is a high demand for collagen and its derivatives, because of their bioactive effects in preventing skin aging (e.g., antioxidants, antiglycation, and antityrosinase) [5].

After testing using the DPPH scavenging assay, several types of marine collagen and its hydrolysate were shown to have antioxidant activity. Hydrolysis can potentially reduce the IC50 (concentration at which 50% inhibition is achieved) value of a biomaterial. However, in fact, the IC50 value of each collagen and its hydrolysate may vary depending on the species and organ used. For example, collagen from the skin of *Chirocentrus dorab* has an IC50 value of 926.25 ppm [6]. In comparison, Nurilmala et al. found that collagen from yellowfin tuna (*Thunnus albacares*) skin needs to be hydrolyzed into collagen hydrolysate to reduce the IC50 value from 560.51 ppm to 119.1 ppm [7]. Other materials, such as scales from croceine croaker (*Pseudosciaena crocea*) and sea cucumber (*Acaudina molpadioides*), have also been successfully converted into collagen hydrolysate, with higher IC50 values of 283 ppm and 385 ppm, respectively [8,9].

Other in vitro parameters that need to be measured include antiglycation and antityrosinase. The antiglycation test is based on the nonenzymatic reaction of protein glycation to form nonstable Schiff base complexes. The production of Schiff bases generates reactive carbonyl compounds that interact with the amino groups of intracellular and extracellular proteins to form AGEs (advanced glycation end products). AGEs accumulate with age and destroy collagen-containing tissue. Using the antiglycation test, it can determined whether there is a decrease in AGEs [10]. Based on prior research, collagen from yellowfin tuna skin is unable to inhibit the glycation reaction, so it needs to be hydrolyzed to produce antiglycation activity of 4.36 ± 2.18% [11].

Melanin overproduction, on the other hand, is also a marker of aging. The inhibition of tyrosinase activity is the focus of most techniques for reducing melanin formation. Tyrosinase is an enzyme that accelerates the pigmentation process by converting L-tyrosine to L-3,4-dihydroxyphenylalanine (L-DOPA), and, finally, dopaquinone, resulting in a polymerization reaction that produces melanin [12]. Despite the fact that melanin is a vital molecule for humans, excessive melanin production in the epidermis can lead to major dermatological issues, such as lentigines, melasma, and hyperpigmentation related to skin aging. Therefore, tyrosinase inhibitors have great potential as whitening agents because they stop melanin synthesis by blocking this enzymatic pathway [13]. Meanwhile, antityrosinase tests on fish collagen are still rarely performed. Fauzi recorded that even hydrolyzed and fractionated yellowfin tuna (*T. albacares*) skin collagen could only block tyrosinase enzyme by 45.71% [14]. Thus, the exploration of marine organisms that can aid in the production of bioactive molecules is one of the most stimulating for biotechnology.

One potential source of collagen that has never been studied is the skin of mackerel scad fish (*Decapterus macarellus*). The mackerel scad is one of the dominant small pelagic fish species and has a high economic value [15]. This species is common in tropical and subtropical seas, particularly in areas with more oceanic waters, such as Eastern Indonesia. Mackerel scads can be found all year, either inshore or in the open ocean. They are coastal pelagic fishes that range in size from 9 to 18 inches. This particular species of *Decapterus* is distinguished from others in the genus by its narrow body shape, yellowish tail, and distinct white lining (emphysial membrane) on the inside of its upper lip. Mackerel scads contain 6–15.45% protein, 2–4% carbohydrate, and 3–8.36% fat [16,17]. In the current study, we focused on the extraction, characterization, and antiaging bioactivity test of pepsin-soluble collagen (PSC) and its hydrolysate from mackerel scad fish skin (*Decapterus macarellus* Cuvier, 1883).

## 2. Results

### 2.1. Characterization of Pepsin-Soluble Collagen (PSC) and Hydrolyzed Collagen (HC)

**Figure 1:**
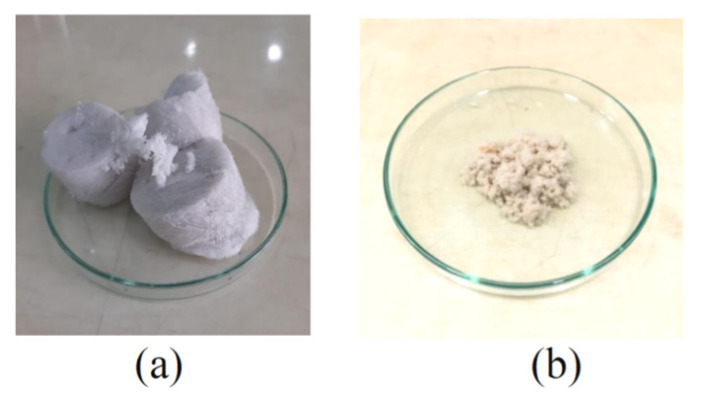
Obtained mackerel scad skin PSC (**a**) and its hydrolyzed collagen/HC (**b**).

Although mackerel scad skin only accounts for a proportion of about 13.04 ± 0.4% of the fish’s weight, PSC extraction was considered easy and efficient. The resulting collagen was a grayish-white (Figure 1a) odorless sponge with a pH of 6.76 ± 0.18, and the final yield was 6.39 ± 0.97% (*w*/*w*). This means that every 100 g of skin (wet weight) could produce 6.39 g of collagen. The resulting yield was 60 times greater than the collagen yield of *Decapterus macrosoma* species, which was found to be only 0.10 ± 0.13% (*w*/*w*) [18]. The main differences with respect to the extraction method in the two *Decapterus* genera above are the pepsin concentration and the duration of immersion. The low pepsin concentration (0.1%) with a longer soaking time (48 h) applied in the current study proved effective for extracting collagen from *D. macarellus* skin. Nevertheless, skin characteristics (i.e., thickness, initial protein content, lysine, and hydroxylysine content) may also influence the yield [19]. Collagen hydrolyzation with 1% collagenase II to produce hydrolyzed collagen (HC) was also quite effective, yielding 96% HC. From 5 g of collagen, 4.8 g of HC were obtained (Figure 1b). The HC obtained product has an agglomerated white powder texture, pH 7 ± 0.16, and is water soluble. The morphology of HC changed drastically from the initial form of PSC, which had a dense, spongy texture. Changes in the morphology of collagen and HC have been found to be common, as has been reported in jellyfish extraction products (*Rhopilema esculentum*) [20] and commercial HC Peptan^®^ F [21].

### 2.2. SEM, SDS-PAGE, and FTIR Analysis

**Figure 2:**
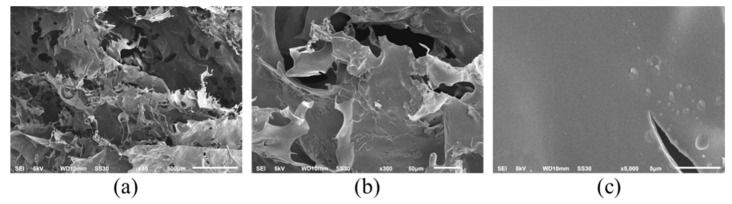
Scanning electron micrograph of mackerel scad skin PSC with different magnification: (**a**) ×50, (**b**) ×300, (**c**) ×5000.

Surface morphological analysis of PSC using Scanning Electron Microscopy (SEM) showed that the collagen looks like a random porous sheet (Figure 2a) and is connected by random-coiled filaments (Figure 2b). At a magnification of 5000×, the collagen surface appears flat, which means that the dehydration process during freeze-drying has been optimal, without causing shrinkage (Figure 2c). The high interconnected porosity of the collagen matrix makes it an excellent scaffold for the growth of fibroblasts and blood vessels, allowing collagen to help repair tissue [22,23].

**Figure 3:**
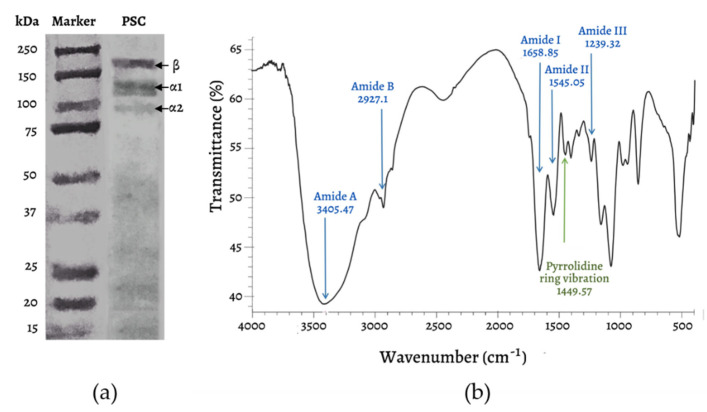
SDS-PAGE analysis of mackerel scad skin PSC (**a**) and FTIR spectra (**b**).

The results of the collagen molecular weight analysis using SDS-PAGE (Figure 3a) showed the typical bands of type I collagen—α1 (~120 kDa), α2 (~100 kDa), and β (~200 kDa) chains. The α1 and β chain bands appear more evident than the α2 band. In previous studies, fish skin collagen has been reported to consist of two or more different chains (heterotrimer—two α1 chains, one α2 chain, and one β chain), and is known as type I collagen. The molecular weight of the α1 chain is around 120–140 kDa, that of the α2 chain around 97–119 kDa, and the β chain around 180–220 kDa [7,24]. In the current study, SDS-PAGE was unable to detect the molecular weight of HC. Small peptides (<20 kDa) tend to bind dyes poorly compared to large peptides [25]. Thus, smaller peptides were harder to detect. Although modifications to the gel percentage and resolving the gel’s pH were made, the molecular weight of HC was still difficult to detect. Therefore, the molecular weight of HC was possibly below the lower limit of the marker band (<15 kDa).

Infrared spectroscopy (FTIR) is an analysis used to determine the functional groups and polypeptide chain configurations of a material related to the properties of molecular bonds and its chemical environment. FTIR spectroscopy can measure the wavelength and intensity of infrared radiation absorption in the sample. FTIR analysis can explore some of the typical chemical characteristics of collagen in the form of five amide peaks, including amide A, amide B, amide I, amide II, and amide III [26], as shown in Figure 3b. The analysis of collagen functional groups using FTIR (Figure 3b) shows that this collagen possesses certain functional groups—amide A, amide B, amide I, amide II, and amide III—which are the typical absorptions of collagen type I. For more details on the collagen peak area, see Table 1.

### 2.3. pH Solubility of Hydrolyzed Collagen

**Figure 4:**
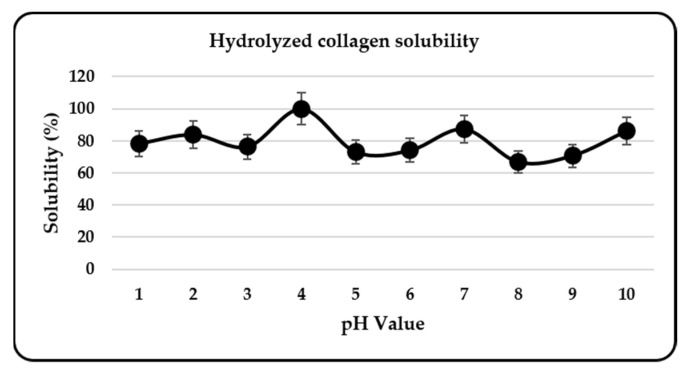
Hydrolyzed collagen solubility.

The solubility of HC from *Decapterus macarellus* was in the range 66.75–100% (Figure 4). The highest solubility of HC was at pH values of 4 (100%), 7 (87.33%), and 10 (86.29%). HC produced with collagenase II hydrolysis demonstrated high solubility throughout the entire pH range (1–10). The high solubility of HC was most likely because of the smaller molecular size of the produced peptides, as well as the release of polar carboxyl and amide groups, which have a high water interaction capacity during the hydrolysis of peptide bonds in the collagen structure. Generally, PSC has a solubility range of 20–100% [18]. PSC can also be more soluble in acidic pH (1–4) and less soluble in iso-electric point (pI) [28]. Compared with PSC, the high solubility of HC in a wide range of pH can be an interesting property, providing the potential for its application in a wide range of pharmaceutical products [11]. As a result of its higher yield and higher solubility over a wide pH range, the effect of HC on DPPH oxidation, glycation, and tyrosinase was evaluated.

### 2.4. Antioxidant Activity of Pepsin-Soluble Collagen and Hydrolyzed Collagen

Antioxidant activity was measured using the 2,2-diphenyl-1-picrylhydrazyl (DPPH) method to determine the IC50 value of PSC and HC. Here, ascorbic acid was used as a positive control. DPPH radicals are deep purple free radicals that require the transfer of electrons or protons (H^+^) from an antioxidant-rich sample; after this, they turn into a yellowish nonradical solution. The IC50 value is the concentration of the test compound needed to inhibit DPPH free radicals by 50%. The IC50 value is obtained from the linear regression of the percent inhibitory activity (y) versus the sample concentration, and is inversely correlated with antioxidant activity.

**Figure 5:**
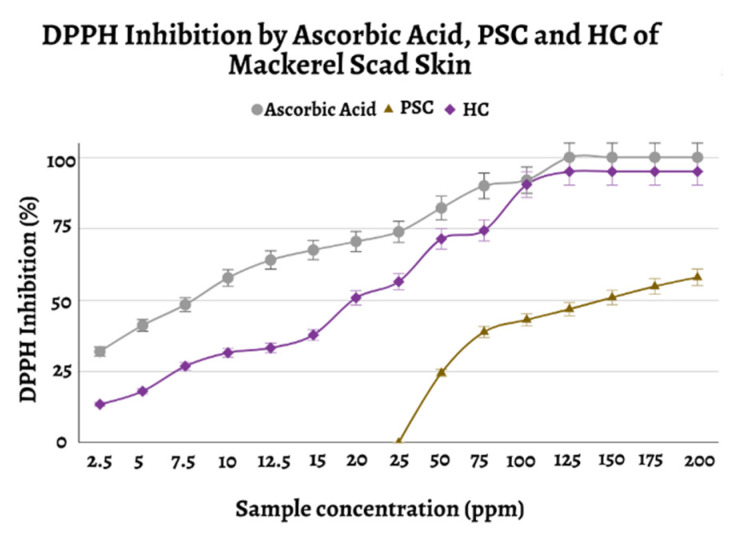
Graph of DPPH inhibition by ascorbic acid, PSC, and HC of mackerel scad skin.

The current study showed that mackerel scad skin PSC had antioxidant activity, with an IC50 of 148.55 ± 3.14 ppm (Table 2; Figure 5). Regarding high-molecular-weight compounds (~100 kDa), this result is superior to previously reported antioxidant activity from fish-derived collagen [7,11,29]. Meanwhile, the IC50 value of HC from *D. macarellus* was found to be 34.966 ± 0.518 ppm. This result is also better than previous results, which found that HC needs to be fractionated with gel filtration chromatography to obtain a lower IC50 value than the extracted HC and collagen [7,11,30].

### 2.5. Antiglycation Activity of Pepsin-Soluble Collagen and Hydrolyzed Collagen

Apart from being an antioxidant, we also demonstrated that mackerel scad skin PSC and HC could prominently inhibit glycation reactions. *D. macarellus* PSC was able to inhibit the glycation reaction by 50% at a concentration of 239.29 ppm. After the collagen was converted into HC peptides, the concentration required for the same inhibitory activity decreased significantly to 68.43 ppm (Table 3). Therefore, in the hydrolyzed form, the antiglycation activity was higher than that of intact collagen. This result is much higher than the antiglycation ability of the HC of *Thunnus albacares*, which is 4.36% at a concentration of 200 ppm [11].

Glycation inhibition can occur through several mechanisms. Aminoguanidine, as a positive control in the current study, is a type B inhibitor that reacts with sugar and diverts it from binding to a protein. Specifically, aminoguanidines react with aldose/ketose carbonyl groups and trap methylglyoxal and 3-deoxyglucosone as the intermediates of AGEs [32].

The mechanism of glycation inhibition by collagen and its hydrolysate competitively inhibits the binding of glucose to proteins by the amino acids it contains. Specifically, glycine—the highest amino acid in collagen—may react with glucose or dicarbonyl derivatives to prevent the formation of a Schiff base, thereby reducing the production of AGEs [33].

### 2.6. Antityrosinase Activity of Pepsin-Soluble Collagen and Hydrolyzed Collagen

The antityrosinase activity of PSC and HC from *D. macarellus* showed a similar trend with the antioxidant and antiglycation data. Kojic acid, which serves as a positive control, demonstrated a four-times-higher inhibitory effect than PSC, and approximately 1.25 times higher than HC. Even so, the inhibitory effect shown in the current study are greater than previous studies on collagen extracted from golden sea cucumber (*Stichopus hermanni*), with an IC50 value of 5610 ppm on the L-DOPA substrate [34]. Table 4 shows that the antityrosinase activity of HC increased threefold compared with the activity of PSC from *D. macarellus*. At a concentration of 79.35 ± 0.5 ppm, HC from *D. macarellus* resulted in a 50% inhibition of tyrosinase activity. This concentration was five times less than the HC of *Thunnus albacares*, which requires a concentration of 400 ppm to inhibit tyrosinase enzymes by 45.71% [14]. Other studies have also reported the ability of collagen peptides isolated from jellyfish (*Rhopilema esculentum*) and cod (*Gadus macrocephalus*), with a tyrosinase inhibitory activity of 50% at concentrations of 78 ppm and 100 ppm, respectively [35,36]. Thus, HC from *D. macarellus* may have strong potential as an antimelanogenic compound.

The value IC50 in the antityrosinase analysis was difficult to compare because of varying test conditions (substrate concentration, incubation time, type of solvent, and source of tyrosinase). The majority of studies using kojic acid, arbutin, or hydroquinone as positive controls produced IC50 values in the range of 55 ppm to 136 ppm. In samples extracted from organisms, a maximum IC50 value of 6300 ppm has usually still been considered significant for inhibiting tyrosinase [37].

## 3. Discussion

In the current study, collagen was successfully extracted using the acid–pepsin method. During immersion, acetic acid further swells the fish skin, interferes with the hydrophobic and electrostatic interactions between the tropocollagen units, and releases collagen [18]. Meanwhile, pepsin acts on the nonhelical peptide chain of collagen and has no effect on the helical peptide chain of collagen (tropocollagen). Pepsin has better reaction selectivity and is less damaging to collagen proteins. Thus, the extracted collagen will have better purity and maintain stable physical and chemical properties [19]. Collagen tends to be grayish-white and odorless. The results of the pH measurements showed that the acidity of collagen was close to neutral, namely pH 6.76 ± 0.18. This result was sufficient compared with the Indonesian National Standard for the collagen pH range, which is 6.5–8 [38]. With these results, the dialysis process using 0.2 M sodium phosphate buffer (pH 8) increased the pH of collagen. Based on the FTIR and SDS-PAGE results, the obtained PSC can be classified as type I collagen. Here, pepsin did not damage the collagen structure, so collagen can be stored for conversion to HC or directly tested for bioactivity.

In the HC extraction process, the chosen protease is the collagenase type II enzyme. The collagenase type II enzyme used in the hydrolysis of collagen *D. macarellus* has high collagenolytic and peptidolytic activity [39]. The fibrillar structure of type I collagen will be cleaved into 3/4 or 1/4 peptide fragments through the mechanism of the collagenase II enzyme [39]. The collagenase II enzyme can break down type I collagen in the glycine-leucine and glycine-isoleucine bonds located at the N end. The hydrolysis of collagen causes the formation of short peptide chains so that many NH3^+^ and COO^-^ groups are formed. [40]. Therefore, it is possible that more exposed hydrophobic residues can form interactions to stabilize the peptide structure.

Before conducting a bioactivity test, it is necessary to know the molecular weight of HC. However, in the present study, the SDS-PAGE analysis did not produce an exact figure for the molecular weight of HC. In the future, further analysis with LC/MS will be carried out so that HC can be further developed for the in vitro analysis of fibroblast cells and wound healing in vivo.

However, HC can meet the solubility characteristics required for the basic ingredients of cosmetic and nutraceutical products, because it has a maximum solubility (100%) at pH 4, which is suitable for skin pH. The hydrolysis of collagen to break down peptides can form stable bonds to increase its solubility. HC with a lower molecular weight has the potential to have better solubility because of the open ends of the amino acids that interact with each other to stabilize the peptide bond [41].

Both the PSC and HC of *D. macarellus* have also shown good antiaging bioactivity. The current study has shown that PSC has moderate intensity of antioxidant activity, while HC can be categorized as a very strong antioxidant. The type, composition, chain length, and sequence of amino acids in the collagen and HC chains affect the ability to fight free radicals [42]. PSC and HC containing phenylalanine and histidine can increase DPPH inhibitory activity, because there is an imidazole ring that acts as a proton donor [43,44]. Furthermore, a proline containing a pyrrolidine ring can act as a hydrogen donor through the carboxyl group to neutralize hydroxyl radicals [45]. Hydrophobic amino acids (glycine, histidine, methionine, valine, asparagine, and glutamine) are thought to affect antioxidant activity [46,47]. Hydrophobic amino acids facilitate the scavenging of DPPH radicals, because they can form beneficial microhydrophobic conditions for peptide molecules. The hydrophobic interactions that occur can place peptides from PSC/HC in the membrane layer during oxidative reactions. This hydrophobic peptide can protect the oxidation of collagen macromolecules by donating protons/electrons to reactive radicals [48]. Lower molecular weight peptides have also been reported to have stronger antioxidant activity [49,50,51]. Most of the antioxidant-powered peptides sourced from organisms have a molecular weight between 0.5 kDa and 1.8 kDa [52]. Generally, these peptides contain <20 amino acid residues per molecule [53]. The lower the molecular weight, the higher the probability that the peptide can cross the intestinal barrier and effectively interact with free radicals to inhibit the propagation cycle of fat peroxidation [54,55].

Mackerel scad skin PSC and HC, which have been proven to have antioxidant activity, can also inhibit glycation reactions, which give rise to AGEs. The inhibitors of AGE products can act as absorbers of carbonyl derivatives, as well as antioxidants or metal ion chelators. Therefore, compounds with antioxidant activity can also inhibit the formation of AGEs [10]. Amino acids can inhibit the glycation reaction. Amino acids such as glycine play a competitive role in inhibiting the optimal binding of glucose to proteins. Glycine bound to glucose or dicarbonyl derivatives can prevent the formation of a Schiff base, thereby causing a decrease in AGEs [32].

Tyrosinase inhibition by kojic acid occurs through dinuclear copper chelation at the active site of tyrosinase and through a decrease in dopaquinone products. Collagen and HC also have similar inhibitory mechanisms. Aliphatic amino acids such as alanine, leucine, valine, methionine, and phenylalanine are thought to inhibit the tyrosinase enzyme [56]. The sulfur atom in methionine can interact with copper ions at the tyrosinase active site. In addition, most polar amino acids, such as arginine and histidine, also contribute to metal chelating activity. Furthermore, the aromatic ring on the amino acid helps bind the tyrosinase active site to interfere with the attachment of the enzyme to the substrate [57].

These results are intriguing, considering the rarity of studies that reveal some antiaging bioactivities in marine collagen and its hydrolysate at the same time. Taken together, the results suggest that the skin of mackerel scad fish has potential antiaging properties and can be further developed for pharmaceutical and cosmetic purposes.

## 4. Materials and Methods

### 4.1. Pretreatment

The mackerel scad fish (*D. macarellus*) were obtained from the local market in Surakarta, Indonesia. The scales were removed, washed in running water, and skinned manually using knives. The skin of the mackerel scad fish was cut in cubes of 1 × 1 cm^2^ and soaked in a 0.1 M NaOH solution with a ratio of 1:10 (*w*/*v*) for 6 h at 4 °C. The NaOH solution was replaced every 2 h.

### 4.2. Pepsin-Soluble Collagen (PSC) Extraction

The PSC were extracted by the methodology described elsewhere [26] with modifications. Soaked fish skin in NaOH was removed and neutralized by running water for 15 min or until the pH was neutral. The skin was further soaked with 0.5 M acetic acid containing pepsin (EC 3.4.23.1; powderized; ≥ 500 Units/mg solid, Sigma-Aldrich, St. Louis, MO, USA), as much as 0.1% (*w*/*w*) with a 1: 8 (*w*/*v*) ratio, and stirred for 48 h at 4 °C. The filtrate was filtered and centrifuged at 4000 rpm and 4 °C for 60 min with Eppendorf 5810R (Eppendorf, Germany) to separate the supernatant. The supernatant was precipitated by adding NaCl to a concentration of 2.6 M for 12 h. The precipitated supernatant was then centrifuged again for 20 min to obtain pellets. The pellets were dissolved in 0.5 M (1: 5 *w*/*v*) acetic acid and placed in the dialysis membrane (Carolina Biological; 12 kDa, cut-off, Burlington, NC, USA) to undergo gradual dialysis. Dialysis I used a 0.2 M sodium phosphate buffer (pH 8) for 24 h. Dialysis II used distilled water for 24 h. The dialysate obtained was then dried using a freeze dryer (Telstar^®^ LyoQuest Plus Lyophilizers, Barcelona, Spain). The PSC yield was calculated based on freeze-dried collagen weight compared with the initial wet weight of mackerel scad skin.

### 4.3. Hydrolyzed Collagen (HC) Extraction

HC hydrolysis refers to the modified protocols of [20]. One gram of PSC was suspended in 200 mL of ultrapure water and submerged in a water bath at 37 °C. Then, collagenase type II (2–28–100MG-PW; powderized; ≥125 Units/mg solid, Sigma-Aldrich, St. Louis, MO, USA), here as much as 6250 U/g, was added and stirred for 5 h. The solution was heated at 95 °C for 10 min to terminate the enzymatic reaction. The solution was then stored at room temperature (25 °C) and centrifuged (3000 rpm) for 30 min. The supernatant was freeze-dried for 90 h to obtain HC and stored at 4 °C for further analysis. HC yield was calculated based on the HC weight compared with the weight of PSC.

### 4.4. Scanning Electron Microscopy

PSC was sprinkled on the specimen holder of SEM (JEOL JSM-6510LA, JEOL, Tokyo, Japan), covered with double sticky tape, and then cleaned with a hand blower to remove impurities. Then, the sample was coated with 400 Å of gold palladium with a JFC-1100 Sputter ion machine. The coating was necessary to turn the test object into an electrical conductor to be photographed. The gold palladium-coated sample was placed into the specimen chamber on the SEM machine at a magnification of 50 to 5000 times with a working distance of 6–10 mm at 4.0–5.0 kV. The source of electrons was emitted toward the sample to scan the sample’s surface, and then, gold, as a conductor, reflected electrons to the detector. The scan results were then forwarded to the detector lens.

### 4.5. Sodium Dodecyl Sulfate-Polyacrylamide Gel Electrophoresis (SDS-PAGE)

SDS-PAGE analysis was performed with reference to the method described in [7], but with modifications. The resolving gel and stacking gel used were 15% and 3%, respectively. A total of 2 mg of PSC and HC samples were separately dissolved in 1 mL SDS 5%, incubated at 85 °C for 1 h, and centrifuged at 12,400× *g* for 5 min. The supernatant (20 μL) was combined with Laemmli 2× sample buffer and heated at 85 °C for 10 min. Subsequently, 15 µL of the sample was fed into the well, and electrophoresis was run at 100 V, 13 mA for 3 h. Electrophoresis was stopped when the front dye reached 0.5 cm from the base of the gel. As a protein marker, Bio-Rad Precision Plus Protein^TM^ Standard (15–250 kDa) was used. The gel electrophoresis was stained with a staining solution for 1 h and then fixed with a destaining solution until the gel was clear and protein bands visible.

### 4.6. Fourier Transform Infrared Spectroscopy (FTIR)

PSC (0.1–2% by weight) was pounded together with KBr and pressed at 8–20 tons pressure to obtain a pellet form. In a dry state, KBr was pounded under an infrared lamp to prevent condensation of steam from the atmosphere and then vacuumed to release water. The pellet sample was inserted into the sample holder on an infrared spectrometer, and the spectrum was read through a computer monitor.

### 4.7. HC Solubility Test

HC solubility was tested using pH values of 1–10. A total of 240 mg of collagen peptide was dissolved in 80 mL of 0.5 M acetic acid at 4 °C for 12 h. A total of 8 mL of the sample was poured into a centrifuge tube, and the pH was adjusted in the range of 1 to 10, here by using 1 M HCl or 1 M NaOH. The volume was made up to 10 mL using distilled water. The solution was homogenized at 4 °C for 30 min and then centrifuged at 10,000× *g* at 4 °C for 30 min. A total of 2 mL of the supernatant was poured into a test tube, and 8 mL of Bradford’s reagent was added. Solubility was calculated using a biophotometer (Eppendorf, Germany) at a wavelength of 595 nm.

### 4.8. DPPH Scavenging Activity

The DPPH assay was performed according to [58] by mixing 500 µL of the DPPH solution (Sigma Aldrich, St. Louis, MO, USA) with 500 µL of the antioxidant solution (PSC/HC/ ascorbic acid). The mixture was reacted for 30 min in the dark at room temperature, and absorbance was measured at *λ* = 517 nm using a UV-VIS spectrophotometer (Hitachi, Japan). All the experiments were performed in triplicate, and the scavenging activity (%) was calculated as 100% × (As−Ax)/Ac, where As is the absorbance of the sample + DPPH, Ax is the absorbance of the sample + DPPH solvent, and Ac is the absorbance of the DPPH solvent + DPPH. The IC50 value expresses antioxidant activity, representing the concentration of the test sample that provides a 50% reduction of free radicals from the equation generated by linear regression of percent inhibition.

### 4.9. Antiglycation Activity

The measurement of antiglycation activity refers to [59] but with some modifications. First, an aminoguanidine HCl solution was prepared on PBS (pH 7.4) and diluted to various concentrations of 40, 80, 160, 200, 280, 360, and 400 ppm. Second, PSC and HC solution were prepared on PBS (pH 7.4) separately with a concentration range of 25–200 ppm. The test solutions were prepared, as described in Table 5. All test solutions were incubated at 60 °C for 72 h. After incubation, solutions A and C were added to 30 µL of TCA 100% (*w*/*v*) to stop the glycation reaction. All solutions were incubated again for 10 min at 4 °C. Solutions A and C were centrifuged at 4000 rpm for 13 min at 4 °C to extract the pellets. Each pellet was then dissolved in 1.2 mL PBS until homogeneous. Finally, the entire solution (A–D) was measured for its absorbance at a wavelength of 350 nm.

### 4.10. Antityrosinase Activity

The determination of the tyrosinase enzyme inhibitory activity refers to [13] with modifications. The assay consists of the tyrosinase enzyme (EC 1.14.18.1; lyophilized powder; ≥1000 unit/mg solid, Sigma-Aldrich), L-DOPA as a substrate, and kojic acid as the positive controls. First, a solution of kojic acid was prepared in a phosphate buffer (pH 6.5) from a concentration of 7.8, 15.63, 31.25, 62.5, and 125 ppm. Then, 1000 ppm of PSC and HC stock solutions were separately diluted in a phosphate buffer (pH 6.5) to obtain a solution with concentrations of 100, 150, 200, 250, 300, and 350 ppm. As much as 350 μL of each sample was taken and added to 300 μL of tyrosinase solution. The plates were incubated at room temperature for 5 min and 550 μL of 2 mM L-DOPA substrate was added; they were then reincubated for 30 min at room temperature. The absorbance was measured at λ = 492 nm using a UV-VIS spectrophotometer (Hitachi, Tokyo, Japan).

### 4.11. Data Analysis

The data obtained in the present study were tabulated, and the average and standard error of mean (mean ± SEM) per test parameter were calculated.

## 5. Conclusions

This study demonstrated the extraction, physicochemical characterization, and antiaging properties of pepsin-soluble collagen (PSC) and its hydrolyzed form (HC) derived from the skin of mackerel scad, a pelagic fish primarily inhabiting subtropical and tropical oceanic water. The resultant HC exhibited greater antioxidant, antiglycation, and antityrosinase activities compared to the PSC counterparts. These findings open up new possibilities for developing functional ingredients for nutraceutical and cosmetic. In future, it would be of great interest to further elucidate the antiaging potential of PSC and HC using in vivo platform to assess their physiological role.

## Figures and Tables

**Table 1:** Peak areas of mackerel scad skin PSC.

Peak	Wavenumber (cm^−1^)	Reference (cm^−1^)	Note [26,27]
Amide A	3405.47	3440–3300	N–H stretching
Amide B	2927.10	3080–2889	CH_2_ asymmetrical stretch
Amide I	1658.85	1700–1600	C=O stretching
Amide II	1545.05	1580–1500	N–H bending
Amide III	1239.32	1350–1200	N–H bending and C–N stretching

**Table 2:** Antioxidant activity of the ascorbic acid, PSC, and HC of mackerel scad skin.

Sample	IC50 (ppm)	Category [31]
Ascorbic Acid	8.61 ± 0.26	Very Strong (IC50 < 50 ppm)
PSC	148.55 ± 3.14	Average (100 < IC50 < 150 ppm)
HC	34.966 ± 0.518	Very Strong (IC50 < 50 ppm)

**Table 3:** Antiglycation activity of aminoguanidine, PSC, and HC of mackerel scad skin.

Sample	IC50 (ppm)
Aminoguanidine	42.78 ± 0.54
PSC	239.29 ± 15.67
HC	68.43 ± 0.44

**Table 4:** Antityrosinase activity of the kojic acid, PSC, and HC of mackerel scad skin.

Sample	IC50 (ppm)
Kojic Acid	66.06 ± 0.63
PSC	234.66 ± 0.185
HC	79.35 ± 0.5

**Table 5:** Component of antiglycation assay.

Materials	Solution A (Glycation Control) (µL)	Solution B (Control Blank) (µL)	Solution C (Sample) (µL)	Solution D (Sample Blank) (µL)
BSA	500	500	500	500
PBS	100	500	-	400
Glucose	400	-	400	-
PSC/HC/Aminoguanidine	-	-	100	100

## Data Availability

The data presented in this study are available on request from the corresponding author.
